# Recruiting and engaging new mothers in nutrition research studies: lessons from the Australian NOURISH randomised controlled trial

**DOI:** 10.1186/1479-5868-9-129

**Published:** 2012-10-29

**Authors:** Lynne A Daniels, Jacinda L Wilson, Kimberley M Mallan, Seema Mihrshahi, Rebecca Perry, Jan M Nicholson, Anthea Magarey

**Affiliations:** 1 School of Exercise and Nutrition Sciences, Institute of Health and Biomedical Innovation, Queensland University of Technology, Brisbane, 4059, Australia; 2 Flinders University, Nutrition & Dietetics, School of Medicine, Adelaide, 5042, Australia; 3 Nutrition Unit, Public Health Group, School of Population Health, University of Queensland, Brisbane, 4072, Australia; 4 Parenting Research Centre, 232 Victoria Parade, Melbourne, 3002, Australia; 5 Centre for Learning Innovation, Queensland University of Technology, Brisbane, 4059, Australia

## Abstract

**Background:**

Despite important implications for the budgets, statistical power and generalisability of research findings, detailed reports of recruitment and retention in randomised controlled trials (RCTs) are rare. The NOURISH RCT evaluated a community-based intervention for first-time mothers that promoted protective infant feeding practices as a primary prevention strategy for childhood obesity. The aim of this paper is to provide a detailed description and evaluation of the recruitment and retention strategies used.

**Methods:**

A two stage recruitment process designed to provide a consecutive sampling framework was used. First- time mothers delivering healthy term infants were initially approached in postnatal wards of the major maternity services in two Australian cities for consent to later contact (Stage 1). When infants were approximately four months old mothers were re-contacted by mail for enrolment (Stage 2), baseline measurements (Time 1) and subsequent random allocation to the intervention or control condition. Outcomes were assessed at infant ages 14 months (Time 2) and 24 months (Time 3).

**Results:**

At Stage 1, 86% of eligible mothers were approached and of these women, 76% consented to later contact. At Stage 2, 3% had become ineligible and 76% could be recontacted. Of the latter, 44% consented to full enrolment and were allocated. This represented 21% of mothers screened as eligible at Stage 1. Retention at Time 3 was 78%. Mothers who did not consent or discontinued the study were younger and less likely to have a university education.

**Conclusions:**

The consent and retention rates of our sample of first time mothers are comparable with or better than other similar studies. The recruitment strategy used allowed for detailed information from non-consenters to be collected; thus selection bias could be estimated. Recommendations for future studies include being able to contact participants via mobile phone (particularly text messaging), offering home visits to reduce participant burden and considering the use of financial incentives to support participant retention.

**Trial registration:**

Australian and New Zealand Clinical Trials Registry Number ACTRN12608000056392

## Introduction

Although reporting of the recruitment and retention of participants in randomised trials has improved with the advent of the CONSORT Statement [1-3], detailed protocols and the lessons learnt are rarely described [4]. Failure to meet recruitment targets can lead to an underpowered study where important and relevant differences between the groups appear to be non-significant, at least in statistical terms. Inadequate recruitment may necessitate an extension of the recruitment period, presenting economic and logistical challenges. A review of 114 trials reported that only 38 (31%) achieved their original recruitment target [5]. When consent rates are low and/or loss to follow up and withdrawals result in high attrition, the potential for *selection bias* and *retention bias* is increased, respectively. Both biases limit the generalisability of results, and reduce the successful translation of any positive outcome effects into practice [6].

A review of randomised trials that included 27 trials which explicitly employed an activity intended to improve *recruitment* found that telephone reminders to non-respondents, use of opt-out rather than opt in consent, and open designs, where participants knew and/or could choose which treatment they were receiving, were particularly successful strategies [7]. Strategies that involved modifying the quantity of, or way that information about a trial was presented to participants did not appear to affect recruitment. However, most of these recruitment strategies need careful consideration, as they also have disadvantages. For example, opt-out trials are controversial from an ethical perspective. In open designs participants and researchers are not blinded to the intervention and measurement of outcomes which may introduce bias. Participants who choose which intervention they receive are likely to have better adherence to that intervention; consequently the effectiveness of the program as a population-based intervention may be markedly overestimated.

In a systematic review of the effectiveness of *retention* strategies in population-based cohort studies, three categories of strategies used were identified: (i) incentives (e.g. financial, non-cash gifts); (ii) reminders, repeat visits or questionnaires, and (iii) other methods (e.g. using certified mail, hand addressing envelopes, reducing questionnaire length) [8]. Use of incentives, or increase in the value of incentives, was associated with increased retention by 2% to 13%. On average, sending reminder letters or repeat questionnaires increased retention by 12% and making reminder telephone calls by 5%. These strategies increase the cost of studies and a clear evidence-base for the effectiveness of each in terms of both impact on recruitment targets as well as overall bias and subsequent generalisabilty is required.

In randomised trials, the inclusion of a placebo arm may reduce patients’ willingness to participate because of the knowledge that they may not benefit directly from the study [9]. Conversely, intensive interventions which involve considerable participant burden may also impact negatively on willingness to participate. To improve study quality it is important that the problems associated with participation in randomised trials are described and quantified and strategies to overcome the barriers are evaluated. Evidence regarding what is realistic in terms of recruitment and retention and which strategies are likely to be effective will strengthen study design, costing and funding applications.

Early feeding interventions for parents offer an opportunity to promote the development of healthy child eating habits and growth during the first two years of life and beyond [10]. However, obesity prevention interventions for children aged younger than two years are not common. A recent review [11] identified 10 studies; most had very small samples and only three were randomised controlled trials (RCTs). Dropout rates across studies were substantial and highly variable (median 32%, range 7–74%) but were independent of study location (home, clinic, classroom, community) [11]. The review did not address reasons for withdrawal but it is possible that factors such as return to work (following parental leave), sick child/family members, transport, and sleep/feeding schedules may negatively impact on both recruitment and retention of parents of young children. NOURISH is a RCT that evaluated the efficacy of a community-based intervention for first-time mothers which commenced when their infants were approximately four to six months of age. The intervention promoted protective infant feeding practices as a primary prevention strategy for obesity in children [12].

This report aims to describe and evaluate the recruitment and retention strategies used in the NOURISH trial in order to inform and support planning for future studies. A major strength of the NOURISH trial is that extensive demographic data on the source population for recruitment were collected, enabling assessment of bias in the sample in terms of *both* selection and retention.

## Methods

NOURISH was an Australian RCT administered by Queensland University of Technology (QUT) in Brisbane, Queensland, and Flinders University in Adelaide, South Australia. The methods used have been detailed in the NOURISH protocol paper [12]. A brief overview is given here, with a more detailed description of methods directly relevant to this paper provided below. First-time mothers were first approached after delivery in the major maternity hospitals in each city. Mothers were later recontacted and consenting mothers and their infants were randomly allocated to either the intervention or control group following baseline measurements (Time 1) when the infants were 2–7 months of age. Follow-up measurements were conducted at two points: Time 2 (infants aged ~14 months) and Time 3 (infants aged ~24 months). Follow-up was six months after completion of each of the two intervention modules. At all three time points data collected included maternal and child weight and height/length measured by study staff at study-specific measurement clinics or home visits, self-reported maternal and infant behaviours assessed by questionnaire and infant food intake assessed by a telephone 24-h recall and two 24-h food diaries.

The NOURISH intervention comprised a comprehensive skills-based program that used a cognitive behavioural approach and focused on the feeding and parenting practices that mediate children’s early feeding experiences. The intervention was delivered via two modules: modules 1 and 2 commenced when the children were approximately 4–6 and 13–16 months of age, respectively and were delivered over 12 weeks. Each module comprised six interactive group sessions (40 groups across both modules and sites) of 1–1.5 h duration. These sessions were co-led by a dietitian (n=13) and psychologist (n=13) at community child health clinics to reduce implementation costs, provide participants with convenient local access and engage the child health nurses. Facilitators received standardised training, used a comprehensive facilitator manual and standard presentation materials, and participated in fortnightly supervision teleconferences to promote intervention quality and integrity. The format was consistent with child health service delivery models at the time in Queensland and South Australia. The control group received self-directed access to usual community child health services that at mothers’ initiative potentially included child weighing, individual appointments with a child health nurse or access to information via a web site or a telephone help line.

Approval for NOURISH was obtained from 11 Human Research Ethics Committees (HRECs) that covered both universities and all the recruitment hospitals (QUT HREC 00171 Protocol 0700000752) and the trial was registered with Australian New Zealand Clinical Trials Registry (ACTRN12608000056392).

### Recruitment

A two-stage recruitment strategy (referred to as Stage 1 and Stage 2) was used to access a consecutive sample of first-time mothers. Eligible mothers who had delivered a healthy term infant (>35 weeks, >2500g) were approached whilst still in hospital (Stage 1) and consent sought for later contact. In Australia 99% of births occur in a hospital or associated birthing centres [13] and a comprehensive or universal home visit program was not available in either city. Mothers were primiparous, aged at least 18 years, with no documented history of domestic violence or intravenous drug use, or self reported eating or psychiatric disorders. Competent written and spoken English, and the ability and willingness to attend sessions at designated venues (in the event of being allocated to the intervention) were also eligibility requirements. The NOURISH intervention was conceptualised as a universal rather than targeted nutrition intervention and hence the selection criteria were designed to identify healthy mothers and babies for whom a community-based group intervention would be suitable.

Due to state-specific ethical and hospital requirements, mothers were approached by hospital midwives in Brisbane and research staff and students in Adelaide. The intention was to screen all women who delivered, including on weekends, and approach all mothers who met the selection criteria. The study was described to the mothers verbally and via a pamphlet. Mothers were invited to provide details for later contact regarding enrolment in the study. Those who agreed to later contact completed a consent form and completed a three page questionnaire; providing demographic data as well as basic information on lifestyle, health and tobacco and alcohol use during pregnancy, self reported pre-pregnancy weight status, intended feeding method and details of two alternative contacts. These mothers were provided with a NOURISH branded folder that contained the information pamphlet and a change of address card. Those who did not agree to be recontacted were asked to provide a sub-set of the same demographic data, as well as their self-reported pre-pregnancy weight status and intended feeding method.

Mothers who gave consent at Stage 1 were recontacted via mail when their infant was aged approximately 4 months (Stage 2). The mail-out was posted in a NOURISH-badged envelope and comprised a (i) personalised cover letter, (ii) four page participant information statement (extended version of the pamphlet provided in hospital), (iii) two page consent form, (iv) green coloured data collection form for those wishing to consent including a checklist of infant health problems and a mental health screener (the Kessler 10 [14]) to assess eligibility, (v) peach coloured data collection form for completion by non-consenters, asking their reasons for non-consent and five questions regarding current feeding, (vi) form for consenters to indicate preferences for the venue, day and time for baseline measurement, (vii) change of address card, and (viii) reply-paid envelope. The cover letter explained the colour coding of the forms, and advised mothers that they had equal chance of winning a $AU50 baby product voucher irrespective of whether they returned a completed green (consent) or peach (non-consent) form. At least three attempts were made by telephone, email, and text messaging to contact mothers who did not respond to the mail-out. Forms were re-sent to mothers who reported they had mislaid or not received the Stage 2 mail. Potential participant dyads were required to be still living locally (i.e. within travelling distance of group sessions), with no serious infant health problems, and a score on the Kessler 10 Psychological Distress Scale (K10; [14]) under 30 (not indicative of high maternal psychological distress).

### Rationale for recruiting two cohorts

Two waves of recruitment occurred, resulting in two participant cohorts. Recruitment of Cohort 1 took place at four Adelaide hospitals and three Brisbane hospitals between February and June 2008. The second wave of recruitment (Cohort 2) took place at two of the same hospitals in Adelaide and one additional hospital (September 2008 – March 2009) and at one of the same hospitals in Brisbane (January – April 2009). From a small pilot (N=105), we anticipated a 60% Stage 1 consent rate, with 70% of these converting at Stage 2 to full enrolment in the trial. For Cohort 1, we exceeded this at Stage 1 with 74% consenting for re-contact (Brisbane: 74%, Adelaide: 72%), but the Stage 2 conversion rate to enrolment (including non-consent as well as those who became ineligible and those unable to contact) was only 31% (Brisbane: 35%, Adelaide: 25%). As a result, Cohort 1 delivered 53% of our recruitment target (N=830), raising concerns about statistical power. Ethical approval was gained for a second round of recruitment (referred to as Cohort 2) at three hospitals in Adelaide (two of the same and one additional) and at one Brisbane hospital. The decision to use only a subset of original hospitals for Cohort 2 recruitment was based on response rates from Cohort 1 which were, at least in part, related to the size, level of engagement and research culture and infrastructure of the hospitals. The Stage 1 consent rate for Cohort 2 was 79% (Brisbane: 88%, Adelaide: 71%).

Other than use of a subset of the original hospitals as outlined above, recruitment procedures for Cohort 2 did not differ from Cohort 1. The exception was an additional mail-out to Cohort 2 mothers when infants were approximately six weeks old (compared with first re-contact at approximately 4 months for Cohort 1). It was hoped that this intermediate contact would improve Stage 2 conversion rates, however the Stage 2 conversion rate (including non-consent, ineligible and unable to contact) of 35% for Cohort 2 (Brisbane: 38%, Adelaide: 31%) was still well below expectations. The mail-out reminded mothers of the study aims and that they would be re-contacted in the next 4–6 weeks. Material mailed included the first NOURISH newsletter, a magnet with the study logo and contact details, and a Stage 2 non-consent form with reply paid envelope. A page of testimonials (de-identified but with permission) from mothers already in the study was included. The idea to include these testimonials came from feedback from Cohort 1 mothers who were participating in Module 1 in Adelaide. Cohort 2 received the same Stage 2 documents as Cohort 1. Again, at least three attempts were made to contact mothers who did not respond to the mail-out.

### Retention strategies

A range of retention strategies were used, that can be categorised into three main themes: (i) participant convenience, (ii) keeping in contact and (iii) tangible incentives. The specific strategies used are detailed below:

i*Convenience* strategies were those intended to make participation as easy as possible. For instance, all mothers were able to select the health care clinic location, day and time most convenient for them to attend. Appointment confirmation, maps and information regarding parking, the questionnaire and, where necessary, a consent form were provided by mail. Mothers were sent text message reminders (via MessageNet; http://www.messagenet.com.au) regarding measurement appointments. In addition, home visits to conduct anthropometric measurements were offered to participants with transport or time constraints, both after hours and on weekends. Home visits conducted were *n*=18 (Time 1), *n*=102 (Time 2) and *n*=147 (Time 3). *Convenience* strategies specific to mothers in the intervention group were also used. For instance, mothers in the intervention group were given a card containing details of all six information session dates and times for easy display on a fridge or pin board, and were sent a reminder text message before each session. For most intervention group mothers, the measurement and intervention sessions were at the same venue, but if not maps and parking information were again provided. Free child care was provided at some Module 1 sessions in Adelaide only (as it was freely available at the venue) and at all Module 2 sessions in both cities. Providing this service for Module 2 sessions was anticipated to improve attendance and reduce the distractions that would arise from having young children present.

ii*Keeping in contact* strategies were those intended to facilitate contact. A study-specific mobile telephone and email address were used and monitored several times each day to ensure timely responses to participants. Participants were given postcards and reply paid envelopes to encourage them to notify NOURISH of change of contact details. Mothers who moved away from either city were encouraged to remain in the study, by being sent instructions for measuring their child at home and reply paid envelopes. Alternate contacts were followed up if participant contact was lost, and letters were sent by Registered Mail as a final attempt to reach participants who were difficult to contact. Newsletters were sent quarterly to participants in both the intervention and control group, with content that was deliberately unrelated to nutrition, growth or weight status. Topics included returning to work after having children, introducing a second language, travelling with children, children’s book ideas, and tips for choosing a car seat.

iii*Tangible incentives* included: (i) bibs and magnets with the NOURISH logo, email contact address and telephone number; (ii) donated moisturiser samples for mother and baby, and (iii) plastic measuring spoons that were also intended to be used for completing the food diaries. Teabags were attached with the second questionnaire. Appreciation certificates with space for a child’s hand-print and magnetic photo frames bearing the NOURISH logo were sent to participants still active at Time 3.

### Baseline assessment and allocation

Consenting mother-infant dyads had baseline (Time 1) weight and height/length measurements taken by trained research staff at government child health clinics, when the child was approximately four months old. As discussed above we provided as wide a range of clinic locations, days and times as was feasible within the constraints of staff availability and costs and access to clinic space. From a specified list, mothers chose the clinic, time and date that they wished to attend. A statistician external to the study was provided with a spreadsheet containing identification numbers of mothers who had been measured and the de-identified clinic at which they had been measured. The statistician stratified by the clinic attended, the rationale being that mothers would be most likely to attend the clinic closest to where they lived, and as such the clinic attended would serve as a proxy for socio-economic status. Thus, dyads were randomised in blocks of four within each stratum. Mothers were informed by mail to which group they had been allocated and those in the intervention group were provided with venue information and a schedule of the group sessions. In all communication with mothers the terms ‘information’ and ‘monitoring’ were used to describe the intervention and control groups respectively.

### Data treatment and statistical analyses

**Figure 1:**
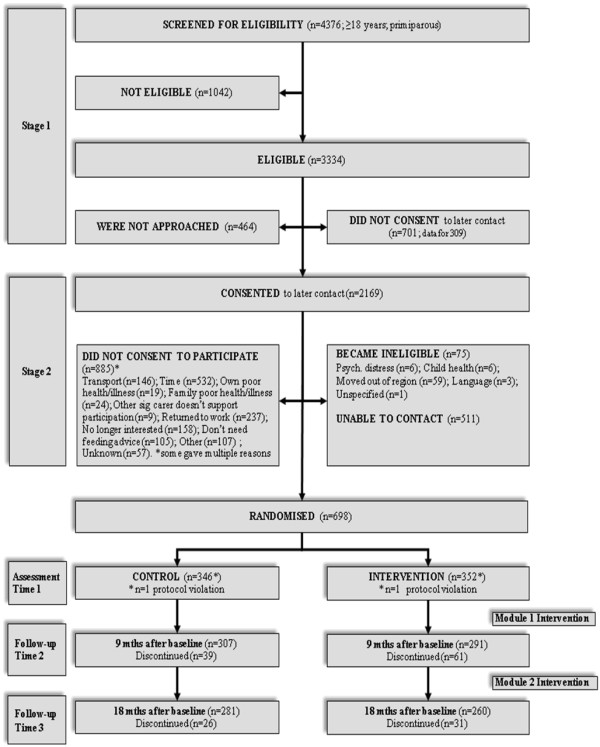
CONSORT diagram showing flow of all participants.

Participants listed in Figure 1 as discontinued at Time 2 either actively withdrew prior to Time 2 or could not be contacted (or did not provide any data) at Time 2 or 3. Those listed as discontinued at Time 3 provided some data at Time 2 but either actively withdrew between Time 2 and 3 or could not be contacted and/or did not provide any data at Time 3.

To assess differences between (i) mothers who participated and mothers who did not; (ii) mothers allocated to control and mothers allocated to intervention group, and (iii) mothers who completed follow-up (Time 3) and mothers who did not, independent samples t statistics and likelihood ratio chi-square statistics were calculated for continuous and dichotomous data collected at Stage 1, respectively. All analyses were conducted using PASW/SPSS Version 18. Level of significance was specified as α=.05 (two-tailed).

## Results

### Response rate

The flow of participants through the study is shown in Figure 1. At Stage 1, of the 3334 mothers screened as eligible, 464 (14%) were not approached, most commonly because of early discharge. Of the 2870 eligible mothers who were approached, 2169 (76%) agreed to later contact. Thus, overall consent rate at Stage 1 was 76%; with consent rates for cohort 1 being similar in Brisbane (74%) and Adelaide (72%) and for cohort 2 in Adelaide (71%); however using only a single hospital in Brisbane for cohort 2 recruitment resulted in a consent rate of 88%. Demographic characteristics of mothers who did not consent at Stage 1 and provided data (*n*=309) and those who consented to recontact at Stage 1 (*n*=2169) are presented in Table 1.

**Table 1:** **Characteristics of *N *=2478 participants who consented (*n*=2169) or did not consent to recontact at Stage 2 (*n*=309)**^**a**^

**Variable**^**b**^	**Consented to recontact (*n*=2169)**	**Did not consent to recontact (*n*=309)**
	**Mean ± SD or %(n)**	
Maternal age at delivery (years)^c^ (*n*=2393)	28.3 ± 5.6	27.1 ± 5.1
Maternal education (University degree)^d^	41 (884)	22 (67)
Born in Australia/New Zealand^d^	76 (1631)	78 (199)
Married/Defacto^d^ (*n*=2389)	90 (1927)	88 (225)
Intend to breastfeed exclusively^d^ (*n*=2457)	91 (1955)	87 (262)

(*n* values) reflect missing data.

^a^ of mothers approached at Stage 1, 702/2871 (24%) declined consent for later contact and of these only 309 agreed to provide brief demographic data.

^b^ based on data provided at Stage 1.

^c^ Mean ± standard deviation (SD) reported.

^d^ % within group (count) reported.

At Stage 2, of the 2169 who consented to recontact, 511 (24%) were unable to be contacted, 885 (41%) did not consent to full enrolment in the study when re-contacted, and 75 (3%) had become ineligible. Reasons for failure to re-contact included disconnected phones, failure to respond to voice messages and incorrect addresses. This resulted in 698 participants consenting to full enrolment. That is, of the 1583 participants who were recontacted and eligible at Stage 2, 44% (*n*=698) consented to participate in the study. The two most common reasons for non-consent to enrolment at Stage 2 (Table 2) were time (60%) and return to work/study (27%). Lack of interest (19%) and no need for feeding advice (12%) were cited less frequently.

**Table 2:** **Reasons given for non-consent at Stage 2 (*n*=823)**

**Reason**	**Frequency**^**a**^
Time	531
Returned to work and/or study	243
No longer interested	159
Transport	149
Do not need advice on feeding	106
Family poor health	24
Other	19
Maternal poor health	19
Temporarily unavailable for sessions	15
Issues with intervention intensity/delivery/follow-up	11
Other significant carer does not support participation	9
Family issues	8
Current or recent participant in another research study	5

^a^ 823/855 (93%) of non-consenters provided at least one reason; 46% respondents provided more than one reason; no data were available for the 511 who could not be contacted at Stage 2.

### Selection bias and sample characteristics

To assess selection bias we compared mothers who consented to participate in the study (at Stage 2) with those who either did not consent or who could not be recontacted at Stage 2 (Table 3). Information collected at Stage 1 was used for these comparisons. Compared to those who either declined consent or could not be recontacted, mothers who consented to participate were older (*M*=30.1, *SD*=5.3 vs. *M*=27.4, *SD*=5.6; *p*<.001), more likely to have completed a university degree (58% vs. 33%; OR=2.9; CI95%=2.4 to 3.5; *p*<0.001), and more likely to have a spouse (either married or defacto; 95% vs. 88%; OR=2.5, CI95%=1.7 to 3.6; *p*<.001). Mothers who consented were less likely to have smoked at any time during their pregnancy (93% vs. 89%; OR=0.4, CI95%=0.3 to 0.5; *p*<.001), and were more likely to report that they intended to breastfeed their baby exclusively (88% vs. 75%; OR=1.8, CI95%=1.3 to 2.5; *p*<.001). Three quarters (77%) of mothers who consented to be recontacted were born in Australia/New Zealand with no difference between women who were allocated compared to those who were not (OR=1.1, CI95%=0.9 to 1.4; *p*=0.3). The success of randomisation of mother-infant dyads to the intervention (*n*=352) or control group (*n*=346) group was checked and no differences on key maternal or infant characteristics between groups were found (data not presented).

**Table 3:** **Characteristics of *N *=2094 mothers who consented at Stage 1 and were Allocated (*n*=698) or Not allocated **^**a**^**(*n*=1396)**

**Variable**^**b**^	**Allocated (*n*=698)**	**Not allocated (*n*=1396)**^**a**^		**Difference **^**c **^***p *value**
	**Consented**	**Did not Consent (*n*=885)**	**Could not recontact (*n*=511)**	
	**Mean ± SD or %(n)**			
Maternal age at delivery (years)^d^ (*n*=2087)	30.1 ± 5.3	28.0 ± 5.5	26.2 ± 5.5	<.001
Maternal education (University degree)^e^ (*n*=2078)	58 (406)	36 (311)	27 (137)	<.001
Born in Australia/New Zealand^e^	78 (542)	77 (667)	75 (376)	.3
Married/Defacto^e^ (*n*=2062)	95 (659)	90 (778)	83 (421)	<.001
Intend to breastfeed exclusively^e^ (*n*=2088)	93 (652)	90 (794)	87 (441)	<.001
Smoked during pregnancy^e^ (*n*=2081)	12 (85)	21 (185)	32 (164)	<.001

(*n* values) reflect missing data.

^a^ excluding an additional 75 participants who became ineligible.

^b^ based on data provided at Stage 1.

^c^ comparison between Allocated vs. Not allocated.

^d^ based on independent samples *t* test; Mean ± standard deviation (SD) reported.

^e^ based on likelihood ratio chi-square test; proportion % (count) reported.

### Retention bias

Seventy-one participants actively withdrew at or before Time 2 and a further 41 before Time 3. An additional 29 participants became lost to contact at Time 2 and a further 16 prior to Time 3. Thus a total of 157 (22%) participants had discontinued the study at Time 3 and based on provision of at least some final outcome data the overall retention rate was 78% of allocated participants. Of the 157 participants who failed to complete the study, 64% discontinued at Time 2 and the relative attrition rate from Time 1 to Time 2 was 14% and from Time 2 to Time 3 was 10%. Key reasons for withdrawal were ‘no longer interested’ (*n*=14), returned to work (*n*=11), poor health of child or family (*n*=11), ‘did not need advice’ (*n*=4) and 15 participants moved out of the region. One participant died before Time 2. Twenty-one participants who actively withdrew did not give a reason. Four participants moved from one study city to the other and continued participation.

More participants from the intervention (*n*=92; 26%) than from the control condition (*n*=65; 19%) discontinued participation in the study (OR=1.5, CI95%=1.1 to 2.2; *p*=.01). Differences between mothers who completed the Time 3 measurements and mothers who discontinued the study are presented in Table 4. Mothers who discontinued participation in the study (Time 3) were younger (*M*=28.0, *SD*=5.5 vs. *M*=30.6, *SD*=5.2; *p*<.001) and less likely to have a university degree (40% vs. 63%, OR=0.4 CI95%=0.3 to 0.6; *p*<.001) than those who completed. Relationship status, smoking during pregnancy, intention to breastfeed exclusively and being born in Australia did not differ between women who completed or discontinued, *p* values ≥0.2.

**Table 4:** **Characteristics of allocated mothers (*N*=698) who Completed the study (*n*=541) or Discontinued (*n*=157)**

**Variable**^**a**^	**Completed (*n*=541)**	**Discontinued **^b^**(*n*=157)**	**Difference *p *value**
	**Mean ± SD or %(n)**		
Maternal age at delivery (years)^c^	30.6 ± 5.2	28.0 ± 5.5	<.001
Maternal education (University degree)^d^	63 (343)	40 (63)	<.001
Born in Australia/New Zealand^d^	81 (438)	82 (129)	.8
Married/Defacto^d^ (*n*=696)	92 (516)	92 (143)	.2
Intend to breastfeed exclusively^d^ (*n*=694)	94 (509)	92 (143)	.3
Smoked during pregnancy^d^ (*n*=696)	12 (62)	15 (23)	.3

(*n* values) reflect missing data.

^a^ based on data provided at Stage 1.

^b^ discontinued due to either active withdrawal or could not be contacted.

^c^ based on independent samples *t* test; Mean ± standard deviation (SD) reported.

^d^ based on likelihood ratio chi-square test; % within group (count) reported.

## Discussion

The NOURISH RCT targeted first-time mothers with young infants; a group likely to face multiple daily challenges (return to work, sick babies/family members, transport, sleep/feeding schedules, etc.) that may negatively impact on their willingness or ability to participate in a research trial. We used a two stage recruitment strategy designed to access a consecutive sample from the major maternity services in the study cities. The rationale was to reduce potential volunteer bias and increase the representativeness of our study sample. Alternative routes of access to a consecutive sample of first-time mothers with infants aged four months, such as through the birth register or the national health insurance database, were not feasible either due to time lags or privacy legislation which prevents disclosure of any contact details without consent. We were able to allocate 44% of mothers who were able to be contacted when their infants were on average 4 months of age, but this represented only 24% of all eligible mothers approached in the maternity hospitals. We may have achieved a similar response rate and a sample with similar selection bias by recruiting a volunteer sample through less expensive approaches such as entirely mail based strategies or advertising through the media, general practitioners or child health clinics. However, such an approach does not allow information to be collected from those who do not agree to participate and limits characterisation of the selection bias.

Comparison on basic demographic characteristics revealed some participation bias as expected in any pragmatic trial that requires substantial active intervention participation and extensive face-to-face assessment. Of the 2169 who consented to recontact at Stage 2, allocated mothers were 2–3 years older, more likely to have a university education, more likely to have a spouse and less likely to have smoked during pregnancy. Similar response rates and/or participation biases are apparent in comparable Australian studies. For example, antenatal recruitment of women with a family history of asthma to an asthma prevention study (CAPS) from six Sydney hospitals in the late 1990s yielded a consent rate of 29% with similar selection bias towards mothers with university education [15]. A recent RCT of an early intervention to prevent childhood obesity gained consent from 25% of eligible first-time mothers approached in antenatal clinics in a disadvantaged area of Sydney [16]. Twenty three percent had completed tertiary education but data have not been published on those who declined to participate. Another recent Australian cluster RCT evaluating an early obesity prevention intervention recruited through pre-existing first-time mothers’ groups set up by randomly selected Maternal and Child Health Centres. Although the consent rate was very high (88%), the rate of tertiary education was very similar to that in our study and no data were available on the estimated third of mothers who do not participate in these groups [17]. Overall, it appears that based on individual-level recruitment, regardless of strategy and general level of disadvantage of the source population, only a quarter of mothers with very young infants will participate in an intervention study. Furthermore, even when recruitment rates are very high selection bias towards more educated mothers remains. It is unclear the extent to which the barriers to participation by less advantaged mothers relate to the intervention itself or participation in research. Regardless, strategies to engage in research, samples that are truly representative of mothers of young infants, remain an important design challenge if study outcomes are to be broadly generalisable.

### Recruitment strategies

On reflection there were a number of aspects of our recruitment protocol that may have contributed to the disappointing consent rates. Informal feedback from participants suggested that being approached whilst still in hospital was probably not an ideal time, due to feeling overwhelmed as a new mother, sleep deprivation, already having multiple forms to complete and generally feeling incapable of processing further new information. The need to rely on hospital midwives in Brisbane rather than study staff, as in Adelaide, may have had an adverse effect as potentially they were not as engaged or as enthusiastic as study staff. Although Stage 1 consent rates were comparable across cities (at least for cohort 1 where a range of hospitals in both Brisbane and Adelaide were used), it is difficult to evaluate whether midwives/study staff improved/worsened response rates due to a range of potential differences between cities (e.g. hospitals, health systems, characteristics of the mothers, socio-economic status, etc.) that may confound such a comparison. From an ethical perspective there are arguments for having consent undertaken by staff independent of the study. In CAPS [15] there did not appear to be any substantive difference in consent rates according to whether initial screening contact was made by midwives, study staff or private clinic receptionists. In CAPS written consent was subsequently obtained by study staff at a home visit but does not appear to have resulted in higher response rates compared to the use of mailed-out questionnaires in the NOURISH trial.

The timing of full enrolment, baseline measurement and intervention session when the infants were approximately 4 months of age may have been a barrier for many women, as it seemed to coincide with them returning to work. This is consistent with lack of time and return to work being by far the most frequently cited reasons for non-consent. It should be noted that at the time of recruitment there was no universal maternity leave payment scheme in Australia and many women may have had limited access to either paid or unpaid leave.

Telephone reminders have been shown to be a successful strategy in increasing recruitment in one study [18], but not in others [19,20]. We are unable to accurately quantify the unprompted response rate to the initial mail-out of 2169 Stage 2 packages but generally it was disappointing. We then attempted to contact the high number of non-responders by telephone which proved extremely time-consuming and, despite three or more attempts (including messages and after-hours calls), we were still unable to contact 24% of potential participants. At Stage 1 we collected alternate contacts but failed to have a system whereby those nominated clearly had permission to provide new contact details and, as a result, this strategy proved to be of limited use. In retrospect, given that we had to telephone so many participants, it may have been more efficient to have undertaken the initial Stage 2 contact by telephone and avoid the considerable cost of printing and posting the packs and time wasted waiting for a mail response. However it was our experience that, even with mobile telephones and email contact details, these mothers were difficult to contact. It is recommended that adequate staff and time for repeat contacts are factored into budgets and time lines. Given the rapidly rising engagement with social networking sites (e.g. facebook, http://www.facebook.com, or linked in, http://www.linkedin.com/), these may provide an effective means of contacting participants.

### Retention strategies

We achieved a pleasing overall retention rate of 78% at infant age two years. This is comparable to the retention rate of 75% at two years of age recently reported for another Australian RCT of an obesity prevention intervention commencing in infancy. [21] A recent pilot study [22] examining an early feeding intervention with first-time mothers (*n*=160) reported 69% retention at age 12-months. In these studies the intervention was delivered via six and two home visits respectively and as such had much lower participant burden than NOURISH. The NOURISH retention rate is consistent with other longitudinal nutrition studies such as the dietary intake and anthropometric sub-studies of the well-regarded ALSPAC which had 5–6 year retention rates of 54% [23] and 64% [24]. Consistent with the pattern of selection bias, there was evidence of attrition (retention) bias in both maternal age and education. The potential impact of this bias on outcomes will be considered through planned comprehensive process and impact evaluations.

The finding that withdrawal from NOURISH was significantly associated with allocation to the intervention group is of interest. The most common reasons for withdrawing - return to work or loss of interest in the study - and the higher proportion of withdrawals from the intervention group may reflect a perception that attending the information sessions was too time-consuming or not important. However, even if the intervention was modified to an at home format (e.g. DVD that mothers could watch at home), there is scarce evidence that this would necessarily improve retention rate. Participants in the Mothers in Motion community-based RCT were overweight and obese women aged 18–34 years who received a DVD for viewing at home that was supplemented with a fortnightly peer support group teleconference [25]. At the end of the 10-week intervention, 48 (41%) participants remained in the study with evidence of retention bias in both age and education similar to that seen in our study. Potential limitations of using a DVD approach for NOURISH include reduced fidelity between the study groups if mothers in the intervention group were to share their DVD with mothers in the control group and difficulty assessing the dose of the intervention received. There would also be less opportunity for face-to-face participant interaction and peer support. It is interesting that despite delivery of the intervention via home visits, the other recent Australian study [21] discussed above showed very similar retention rates to that achieved in NOURISH.

It is difficult to identify which specific retention strategies were most effective, as multiple approaches were implemented simultaneously. A strategy that proved helpful in maintaining and in some cases re-establishing contact was text messaging. This was made convenient and cost effective by using a computer program to send the texts. Two particular benefits are that message retrieval is free for participants (compared to some voice mail facilities), and it is possible to maintain contact with participants who move and do not provide a new landline number or address. Just under three-quarters of participants in one study indicated text messaging was an acceptable form of contact for them [26]. As in NOURISH, text messaging was sometimes the only way by which the researchers could contact some participants.

The increase in home visits requested and conducted across the three time points in both cities suggests that this convenience strategy was well-received and valued by participants. Indeed, 27% of final outcome measurements were done through home visits. Although these were a significant unplanned impost on our budget, they were deemed necessary to optimise our overall retention rate. Home visits are being used in the observational Longitudinal Study of Australian Children (LSAC; *N*=4983) which has retention of 86% over three waves of data collection, to age 4–5 years [27]. An obvious disadvantage to conducting anthropometric measurements at participants’ houses is costs relating to mileage and staff time, and the need for multiple complete sets of measurement equipment.

It is unclear whether offering financial incentives would have encouraged greater retention in NOURISH. A recent systematic review of studies that specifically evaluated the effectiveness of retention strategies concluded that financial incentives were associated with increased retention rates [8]. A financial incentive approach was used in the Making Our Mealtimes Special (MOMS) study [28], an anticipatory guidance program designed to prevent childhood overweight and obesity. Mothers of healthy infants aged two months or younger were approached by study staff in primary care clinics. Following informed consent procedures, participants completed an initial survey and were given a $US10 gift card, magnet with study contact details and a copy of the incentive program for participation (i.e. gift cards increasing in monetary value for completion of each survey). Retention rates at 6 months and 12 months post-recruitment were 75% and 64% respectively. The authors attributed these rates to the range of retention strategies employed, including financial incentives.

As discussed above, the rapidly increasing engagement with social networking sites may be an effective portal for distributing updates and reminders. Some Brisbane participants established their own group on a social networking site, spontaneously and independently from the research team. A limitation of this approach is it may decrease fidelity between the study groups if mothers in the control group also access the site. The internet could also have been used to distribute questionnaires to mothers, as a high number provided their email address. However, collection of anthropometric measures by trained assessors using standard equipment would be preferred over self-report.

## Conclusions

NOURISH is one of the first studies internationally to investigate an obesity-prevention intervention for children younger than 12 months. Completion rates for children enrolled in obesity treatments are low [29], reinforcing the importance of effective primary prevention strategies in order to reduce the demand for treatment. Data from NOURISH will be combined with other similar concurrent studies in a prospective meta-analysis [30], which will further strengthen the value of this study.

The consent and retention rates of our sample of first-time mothers are comparable with or better than similar studies. The resource-intensive recruitment strategy that was designed to access a consecutive sample resulted in a disappointing proportion of eligible mothers entering the trial and significant selection bias. Nevertheless, unlike most other primary prevention interventions, the NOURISH recruitment strategy involved collection of extensive demographic data on the recruitment source population enabling detailed assessment of selection bias. Selection and retention bias related to age and education are common and probably unavoidable in intervention studies, but nevertheless need to be quantified and the implications for findings carefully considered. Thus, despite being resource intensive, the recruitment strategy used had clear benefits. Being able to contact participants via mobile phone (particularly text messaging) appeared to be important for staying in contact. Offering home visits for measurements became critically important for retention of participants in the study. However, despite reimbursement of travel costs and the provision of child care at intervention group sessions, higher withdrawal rates from the intervention group suggest that attending the information sessions placed too high a burden on participants. Retention of intervention group participants may be improved by offering alternatives to attendance at group sessions, and retention of participants from either group may have been improved by offering financial incentives.

## Competing interests

The authors declare that they have no competing interests.

## Authors’ contributions

LD and AM led the design and successful funding application for NOURISH. JW and RP had operational responsibility for recruitment. Analysis was undertaken by JW, KM and SM. The manuscript was drafted by LD, JW and KM but all authors contributed to study design and implementation and/or data interpretation and manuscript preparation. All authors read and approved the final manuscript.
